# Novel P335-like Phage Resistance Arises from Deletion within Putative Autolysin *yccB* in *Lactococcus lactis*

**DOI:** 10.3390/v15112193

**Published:** 2023-10-31

**Authors:** Jenny Seiler, Anne Millen, Dennis A. Romero, Damian Magill, Laura Simdon

**Affiliations:** 1 IFF, Madison, WI 53716, USA; anne.millen@iff.com (A.M.); dennis.romero@iff.com (D.A.R.); laura.simdon@iff.com (L.S.); 2 IFF, 86220 Dangé-Saint-Romain, France; damian.magill@iff.com

**Keywords:** *Lactococcus*, bacteriophage, autolysin, resistance, *yccB*

## Abstract

*Lactococcus lactis* and *Lactococcus cremoris* are broadly utilized as starter cultures for fermented dairy products and are inherently impacted by bacteriophage (phage) attacks in the industrial environment. Consequently, the generation of bacteriophage-insensitive mutants (BIMs) is a standard approach for addressing phage susceptibility in dairy starter strains. In this study, we characterized spontaneous BIMs of *L. lactis* DGCC12699 that gained resistance against homologous P335-like phages. Phage resistance was found to result from mutations in the YjdB domain of *yccB,* a putative autolysin gene. We further observed that alteration of a fused tail-associated lysin-receptor binding protein (Tal-RBP) in the phage restored infectivity on the *yccB* BIMs. Additional investigation found *yccB* homologs to be widespread in *L. lactis* and *L. cremoris* and that different *yccB* homologs are highly correlated with cell wall polysaccharide (CWPS) type/subtype. CWPS are known lactococcal phage receptors, and we found that truncation of a glycosyltransferase in the *cwps* operon also resulted in resistance to these P335-like phages. However, characterization of the CWPS mutant identified notable differences from the *yccB* mutants, suggesting the two resistance mechanisms are distinct. As phage resistance correlated with *yccB* mutation has not been previously described in *L. lactis*, this study offers insight into a novel gene involved in lactococcal phage sensitivity.

## 1. Introduction

*Lactococcus lactis* and *Lactococcus cremoris* represent a diverse group of strains that are used extensively as starter cultures for fermented dairy products. Due to the nature of commercial dairy fermentations, virulent bacterial viruses (bacteriophage, or phage) are commonly found in the industrial environment [1]. If left uncontrolled, phages will interfere with fermentations, resulting in disruption of the process and loss of product quality. Natural mutagenesis to generate bacteriophage-insensitive mutant (BIM) populations has been a standard approach for addressing phage susceptibility in dairy starter strains [2]. However, the resultant mutation(s) can impact genes essential for desirable functionality in milk, often resulting in poor growth and slow acidification [3,4]. 

Lactococcal phages are classified into ten groups [1], three of which have historically been particularly problematic in dairy environments. These include the *Skunavirus* genus (formerly termed 936 group), *Ceduovirus* genus (formerly termed c2 group), and P335 group, with P335 group phages being remarkably diverse and difficult to address [5,6]. Comparative analyses have shown that there is no defined core genome for the P335 phage group; although members are interrelated through certain properties and modules, there is no single attribute shared among the group [7]. P335 phages may be strictly lytic or lysogenic and are described to possess mosaic structures that are presumed to be the result of recombination events between virulent and lysogenic phages [8]. Additionally, in response to host-directed phage resistance, P335 phages can acquire chromosomal or plasmid-encoded genes to circumvent the defensive mechanisms, adding to their diversity [9,10,11]. *L. lactis* and *L. cremoris* prophages are lysogenic phages affiliated with the P335 group [12,13], often referred to as the temperate P335 group or the P335 quasi-species [6,14,15]. However, the overall diversity among P335 phages and propensity for genetic exchange make it difficult to classify newly identified phages and implement effective resistance mechanisms against them. 

Previous reports have identified polysaccharides associated with the lactococcal cell surface as receptors for many P335 phages [16,17]. These include the cell wall polysaccharides (CWPS) encoded by the *cwps* (also referred to as *rgp* [2]) operon and exopolysaccharides (EPS) produced via the Wzx/Wzy-dependent pathway [17,18]. CWPS are involved in cell wall assembly and cell division, and CWPS mutants of *L. lactis* have been shown to exhibit increased levels of phage resistance [16,18,19,20,21,22]. However, consequential cell defects of these mutants, which include impaired growth, cell deformities, and increased sedimentation, commonly hinder dairy fermentation qualities [16,18,19,21,22]. Genetic diversity among *cwps* groups has allowed for their categorization into eight major types and at least 21 subtypes [2]. 

Peptidoglycan (PG) is a polysaccharide cross-linked by peptides that form a mesh-like layer and is the main component of the cell wall in Gram-positive bacteria [23]. Bacteria synthesize a variety of enzymes capable of hydrolyzing their own PG, called peptidoglycan hydrolases (PGH) [24]. There are four different classifications of PGH, dependent on which bond in the PG is cleaved: N-acetylmuramidase, N-acetylglucosaminidase, N-acetylmuramyl-L-alanine amidase, and endopeptidase [23,24]. Acetylmuramidases, commonly referred to as lysozymes or autolysins in bacteria, are largely utilized for cell wall metabolism and peptidoglycan recycling but can also be potentially lethal, resulting in cell lysis [24,25,26,27]. It is usually difficult to assign a distinct function to a specific PGH, as many bacteria possess several PGH that appear to have redundant roles [28]; a previous study [29] identified *L. lactis* strain IL1403 [30] to harbor a total of five bacterial-encoded putative PGH, four of which, including AcmA to AcmD, have all been shown to have the same specificity but different modular structures. 

Lysozymes, a type of endolysin, are also utilized by phages [31]. At the end of the lytic cycle, the host cell wall must be broken down to release new progeny virions. Consequently, phage-encoded lysozymes are used in conjunction with phage-encoded holin to digest the PG layer and lyse their hosts [31,32]. A previous study showed that in the absence of phage lysin during phage infection, pneumococcal autolysin LytA facilitates bacterial lysis and phage progeny release [33]. It was furthermore demonstrated that host-encoded LytA is activated by holin-induced membrane disruption [33], supporting the idea that a host-encoded autolysin can facilitate phage infection. Earlier in the infection process, phage adhesion modules including distal tail protein (Dit), tail-associated lysin (Tal), and receptor binding protein (RBP) are responsible for host attachment in lactococci [17,20]. Tal is additionally involved in digestion of PG to aid in phage entry into the host cell [34,35]. 

In this study, we isolated and characterized spontaneous BIMs of *L. lactis* DGCC12699 that are resistant to P335-like phages. These isolates were found to harbor mutations in the YjdB domain of a putative autolysin gene, designated as yccB in some lactococcal genomes [16]. Complementation with a plasmid encoding the wild-type *yccB* and subsequent plasmid curing were performed in *yccB* mutants to test resultant phage resistance phenotypes. Subsequently, homologous P335-like phage D7893, which is infective on the *yccB* mutants, was isolated. The genetic determinant responsible for D7893 infectivity on the *yccB* mutants was identified using comparative genomics followed by construction of recombinant phage. Additional bioinformatic analyses performed on IFF’s proprietary collection of *L. lactis* and *L. cremoris* strains found *yccB* homologs to be widely distributed and gene structure to be strongly correlated with the strain’s *cwps* type and subtype. Furthermore, characterization of a DGCC12699 CWPS mutant was carried out to compare to the *yccB* mutants, including phage sensitivity spot titers, adsorption assays, and acidification profile testing. These results demonstrated that the two resistance mechanisms are likely distinct, providing insight into a novel genetic factor responsible for phage sensitivity in *Lactococcus*.

## 2. Materials and Methods

### 2.1. Bacterial Strains and Phages

Bacterial strains and phages used in this study are listed in Table 1. Unless otherwise specified, lactococcal strains were grown at 30 °C in sterile 11% *w*/*v* nonfat dry milk (NFDM) or in M17 broth [36] supplemented with 0.5% lactose. Differentiation of fast and slow growth in milk was performed using FSDA II medium [37] with the trimagnesium phosphate substituted with β-glycerophosphate. *Escherichia coli* was propagated aerobically in LB broth (Becton, Dickenson and Co., Franklin Lakes, NJ, USA) at 37 °C. When required, erythromycin (Erm) was added to the media as follows: 5 µg/mL for *L. lactis*; 150 µg/mL for *E. coli*. 

Phages were isolated from industrial whey samples from Europe between 2008 and 2021; single plaque isolates were placed into peptone buffer and kept at 4 °C overnight, from which high-titer lysates were prepared. Preparation of phage lysates was performed as previously described [36]. Briefly, 1% phage stock was added to log-phase cultures, followed by addition of CaCl_2_ to a final concentration of 10 mM. Tubes were incubated at 30 °C until clearing was observed, indicating lysis. High titer lysates of >1 × 10^7^ plaque-forming units (PFU)/mL were passed through a 0.45 µm filter and stored at 4 °C. Phage spot titer and plaque assays were performed with exponentially growing cells on MRS medium. Spot titer assays were performed by adding 100 µL of cells to soft agar overlay (0.5% agar *w*/*v*) containing 10 mM CaCl_2_ and pouring onto a standard MRS base plate. Then, 10-fold serially diluted phage lysates were spotted onto the overlay. Plaque assays were performed by combining 100 µL of cells with 100 µL of serially diluted phage and incubating at 30 °C for 15 min. A total of 3 mL of soft agar overlay with 10 mM CaCl_2_ was added to the mixture and poured onto a base plate. The efficiency of plaquing (EOP) was determined by dividing the phage titer on the test strain by the titer on the fully sensitive host strain. Phage adsorption tests were performed as previously described [38] on MRS medium with cells grown to A600 of 0.5.

**Table 1:** Bacterial strains, bacteriophages (phages), and plasmids.

Biological Material	Relevant Characteristics	Reference
Bacteria		
*Lactococcus lactis*		
DGCC12699	Phage-sensitive parental starter strain (DGCC12699 *yccB* accession number: OR544030)	This study
BIM2	DGCC12699 *yccB* mutant	This study
BIM8	DGCC12699 *yccB* mutant	This study
BIM19	DGCC12699 *yccB* mutant	This study
BIM21	DGCC12699 CWPS mutant	This study
BIM2 + pG9yccB	BIM2 transformant harboring pG9yccB	This study
BIM2 + pG9	BIM2 transformant harboring empty pG9 vector	This study
BIM2ΔpG9yccB	BIM2 + pG9yccB after pG9yccB curing	This study
DGCC12699 + pG9RBP_7893_	DGCC12699 transformant harboring pG9RBP_7893_	This study
*Escherichia coli*		
TG1 RepA^+^	Plasmid propagation host; *supE hsd*∆*5 thi* ∆(*kac-proAB*) F’ (*traD36 proAB*^+^ *lac*I^q^ *lacZ*∆M15) with chromosomal copy of pWV01 *repA*	[39]
Phages		
D6867	Propagation host DGCC12699; P335-like (accession number OR480986)	This study
D7138	Propagation host DGCC12699; P335-like (accession number OR480987)	This study
D7893	Propagation host BIM2; P335-like (accession number OR480988)	This study
D5694	Propagation host DGCC12699; *Teubervirus*	This study
D6867-RBP_7893_	Propagation host DGCC12699; D6867 Tal-RBP recombinant	This study
Plasmids		
pGhost9	Erythromycin resistance, temperature sensitive vector; aka pG9	[40]
pG9yccB	pG9 + wild-type *yccB* from DGCC12699	This study
pG9RBP_7893_	pG9 + 3′ end of D7893 Tal-RBP	This study

### 2.2. BIM Isolation

A single-step phage challenge was performed with DGCC12699 and phage D6867. Briefly, 100 µL of log-phase cells were exposed to 100 µL of serially diluted phage at multiplicities of infection (MOI; number of phages relative to the number of cells) of 1, 10, and 100. Mixtures were incubated at 30 °C for 15 min, and then soft agar overlay containing 10 mM CaCl_2_ was added to the mixture, which was then poured onto a base plate. Surviving colonies were randomly selected and screened based on their acidification on FSDA II medium; colonies that turned the pH indicator plates yellow comparably to parent DGCC12699 after two days of incubation, indicative of similar milk acidification properties, were then placed into milk to confirm their ability to clot overnight. 

### 2.3. PCR, DNA Isolation, Sequencing, and Bioinformatic Analyses

Primers and gBlock™ gene fragments were synthesized by Integrated DNA Technologies (Coralville, IA, USA); primer sequences are listed in Table 2, and the gBlock sequence can be found in Table A1. PCR reactions were performed with GoTaq^®^ Colorless Master Mix (Promega Corp., Madison, WI, USA) or Phusion HF Mastermix (New England Biolabs, Ipswich, MA, USA) according to manufacturer’s instructions. PCR products were purified using Wizard SV Gel and PCR Clean-Up System (Promega Corp., USA). Sanger sequencing was performed on purified PCR products by Eurofins Genomics (Louisville, KY, USA). 

Lactococcal genomic DNA was isolated using MasterPure Complete DNA and RNA Purification Kit (LGC Biosearch Technologies, Madison, WI, USA) according to manufacturer’s instructions; phage DNA was isolated from high titer phage lysate (>1 × 10^9^ PFU/mL) using Invitrogen PureLink Viral RNA/DNA Mini kit (Life Technologies, Carlsbad, CA, USA) according to manufacturer’s instructions. Oxford Nanopore and Illumina sequencing reads were used in hybrid assemblies completed with the Unicycler pipeline [41], which includes miniasm and Racon pipelines and pilon polishing. Bacterial DNA sequences were annotated using PATRIC RASTtk-enabled Genome Annotation Service [42] prior to manual curation. Phage and prophage genomes were annotated using prokka v1.14.5 [43] with manual curation of some genes conducted following BLASTp searches against the nr database. Sequences were analyzed using Geneious Prime v2019.0.1 and v2022.2.1 (https://www.geneious.com, accessed on 24 January 2023). Phage genome map construction and alignments were done using the Easyfig tool V2.2.2 [44]. Alignments were conducted with a minimum window size of 200 base pairs (bp) and cut-off E-value of 1 × 10^−5^ for regions of similarity using the tBLASTx algorithm.

Phylogenetic trees were created in MEGA7 [45] and then manipulated using Interactive Tree Of Life (iTOL) v6 [46]. *cwps* groups and subgroups of each strain were identified by analysis of public lactococcal *cwps* operons.

### 2.4. Protein Modeling

Molecular modeling of proteins was conducted using the AlphaFold package in monomer mode and employing the fully compiled database of PDB structures [47]. Predictions were scored based on the pLDDT criteria to choose the top ranked model. Structural alignments were carried out using the TM-align algorithm [48]. Visualization of models and preparation of publication grade figures was conducted within Pymol V2.4 [49].

### 2.5. yccB Complementation and Phage Recombination

pG9yccB and pG9RBP_7893_ were constructed using NEBuilder HiFi DNA Assembly MasterMix (New England Biolabs, USA) according to manufacturer’s instructions. DNA fragments used for each assembly are listed in Table 3. Assemblies were electroporated into TG1 RepA^+^ as described by Dower et al. [50]. Erm-resistant colonies were screened using PCR to identify transformants that were positive for plasmid uptake (Table 3). Plasmids were purified from TG1 RepA^+^ using the GeneJET Plasmid Miniprep Kit (Thermo Scientific, Waltham, MA, USA). The purified plasmids were introduced into *L. lactis* by electroporation as described by Holo and Nes [51]. Erm-resistant colonies were screened using PCR to identify lactococcal transformants (Table 3). Primers internalErmR-F and internalErmR-R were used for detection of empty vector pG9 in transformants (Table 2).

After complementation, pG9yccB was cured from BIM2 transformants by growing in milk at 37 °C in the absence of Erm for three consecutive nights, subculturing into fresh milk each afternoon. Single colony isolates were tested for loss of Erm resistance, and Erm-sensitive isolates were then screened using PCR to confirm absence of pG9yccB (Table 3). To confirm the chromosomal *yccB* was still truncated, transformants were amplified with primers yccBNested_F and yccBNested_R and Sanger sequencing was subsequently performed using primers yccB_F1, yccB_F2, and yccB_F3 (Table 2).

For phage recombination, gBlock D7893_RBP-Rec, containing the 1 kb region of variability in D7893 and 500 bp adjacent regions homologous to D6867, was assembled into pG9 (Supplementary Figure S1). To isolate recombinant D6867 phage that had exchanged the Tal-RBP region for that of D7893, D6867 was propagated on DGCC12699 + pG9RBP_7893_. Sufficient DNA homology flanking the Tal-RBP region allowed recombination between D6867 and pG9RBP_7893_ to occur via double crossover. Selection of recombinant phage was performed by plaque assaying the propagation on BIM2. To confirm integration of the D7893 Tal-RBP in the recombinant population, Sanger sequencing was performed with primers D6867tal_StartF, D6867tal_F1, D7893talCheck_F, and D6867tal_EndR; initial amplification was performed using D6867tal_StartF and D6867tal_EndR (Table 2).

### 2.6. Acidification Profile

For assessment of acidification properties, overnight 11% NFDM cultures were transferred into M17 broth supplemented with 0.5% lactose. Once the cultures reached an A600 of 0.6, they were inoculated at 0.75% (*v*/*v*) into commercial 1% fat Kemp’s milk. Samples were kept in a 30 °C water bath overnight with pH probes and CINAC software monitoring acidification of each strain every 2 min. Each strain was run in duplicate.

## 3. Results

### 3.1. BIM Isolation and Phage Resistance Characterization

P335-like phages D6867 and D7138 and *Teubervirus* D5694 were isolated against industrial starter strain DGCC12699 from routine testing of commercial whey samples. Following separate challenges of DGCC12699 with D6867, surviving colonies were randomly chosen for further evaluation. Representative BIMs BIM2, BIM8, BIM19, and BIM21 were found to be resistant to P335-like phages D6867 and D7138 in spot titer assays, with EOPs < 1 × 10^−7^ (Table 4). BIM2, BIM8, and BIM19 were fully sensitive to Teubervirus D5694, indicating the mutation may only affect P335-like phages. In contrast, BIM21 showed partial resistance against D5694, as plaques were still produced on BIM21 but at a much lower efficiency than on DGCC12699 (Table 4). Adsorption assays were performed with representative phage D6867 to further investigate the nature of the phage resistance phenotypes. Results showed D6867 adsorbed at a high efficiency (>80%) to BIM2, BIM8, and BIM19 and at a reduced efficiency (<45%) to BIM21 (Table 4). Together, these data indicate at least two distinct mutations affecting different steps of the lytic cycle were likely present among the DGCC12699 BIMs.

### 3.2. Identification of Lactococcal Mutations Conferring Phage Resistance

**Figure 1:**
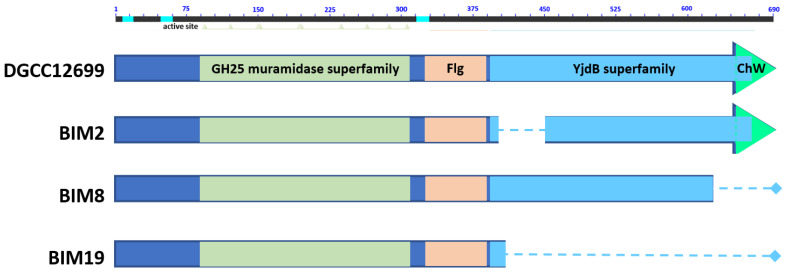
Predicted YccB protein translation comparison. Protein domains found in YccB are shown in different colors and labeled accordingly. Dashed lines indicate protein truncation due to mutation. An amino acid scale is included on top where the active site of the protein is also shown in light green.

To identify the mutation(s) responsible for phage resistance, whole genome sequencing was performed on a representative BIM displaying each distinct phage resistance phenotype (BIM2 and BIM21). Comparison of BIM2 to DGCC12699 revealed an in-frame 153 bp deletion at nucleotide position 1225–1378 in putative autolysin gene *yccB* [2073 bp/690-amino acids (aa)], resulting in an internally deleted protein of 639 aa. This putative autolysin contains a catalytic C-terminal glycosyl hydrolase (GH25) and an immunoglobulin (Ig)-like domain YjdB (Figure 1). Blast analysis identified the gene designated as *yccB* in certain lactococcal strains [IL1403 (AE005176.1 [16]); KF147 (CP001834.1); CV56 (CP002365.1); AI06 (CP009472.1); A12 (LT599049.1)]. Although whole genome sequencing was not performed, *yccB* was sequenced in BIM8 and BIM19, which identified a single nucleotide deletion within a run of G’s at position 1804–1807 and a G→A SNP at nucleotide position 1229, respectively, both resulting in truncation of the gene. In each BIM, the mutation was shown to exclusively affect the predicted YjdB domain, with the predicted muramidase domain upstream unaffected (Figure 1). In contrast, *yccB* was found to be intact in BIM21. Instead, BIM21 was found to harbor a single nucleotide insertion at position 136 of a glycosyltransferase family 2 gene within the *cwps* cluster, resulting in truncation of the gene.

To determine if complementation with the wild-type *yccB* would restore phage sensitivity, pG9yccB, which encodes the full DGCC12699 *yccB* gene sequence, was introduced into BIM2; empty vector pG9 was transformed into BIM2 in parallel. Transformant BIM2 + pG9yccB was found to be fully sensitive to both D6867 and D7138, while transformant BIM2 + pG9 remained phage-resistant (Table 4). DGCC12699 was electroporated with pG9yccB in parallel, however, no transformants were obtained, suggesting that multiple copies of the wild-type gene may be lethal to the cell. Subsequently, pG9yccB was cured from BIM2 + pG9yccB (pG9yccB – cured isolate = BIM2ΔpG9yccB), which restored phage resistance (Table 4). This further confirmed that the wild-type *yccB* is required for phage sensitivity. Sequencing confirmed that the deletion was still present in the BIM2ΔpG9yccB chromosomal *yccB* gene.

### 3.3. Phage Genome Analyses and D7893 Infectivity

**Figure 2:**
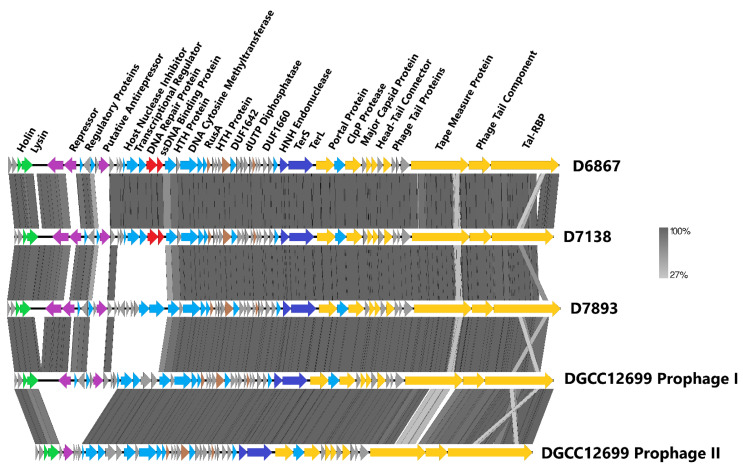
Phage genome alignments. Regions of similarity exhibiting a minimum identity overlap of 200 bp are shown in varying scales of gray according to the percentage identity observed. Genes exhibiting globally related functions possess the same color scheme and are labeled accordingly (yellow = structural, dark blue = DNA packaging, purple = life cycle regulation, red = DNA replication or repair, brown = domain of unknown function (DUF), light blue = other function, gray = hypothetical protein).

The isolation of phages D6867 and D7138 against DGCC12699 prompted its replacement with BIM2 as a commercial starter strain. Subsequently, phage D7893 was isolated against BIM2 during routine testing and was also found to infect parent DGCC12699 (Table 4). Interestingly, BIM21 was found to be resistant to D7893 (Table 4). Genomic analyses showed D6867 and D7138 share ~92% nucleotide identity, while D7893 shares ~80% and ~76% nucleotide identities with D6867 and D7138, respectively. Further analysis revealed D6867 and D7138 share >96% and >76% nucleotide identities with Prophage I and Prophage II in DGCC12699, respectively, suggesting they may have existed in a lysogenic state before becoming virulent or vice versa. An alignment of the phage genomes can be found in Figure 2.

A general BlastN comparison of the virulent phages found partial homologies (covering up to ~50% of the genomes) primarily to *L. lactis* (>90% identity) and unspecified *Caudoviricetes* genomes. P335 group phages have been classified into morphotypes based on morphological characteristics and sequence homology within the adhesion module, which includes the Dit, Tal, and baseplate/receptor binding protein (Bpp/RBP) [13,20]. Genomic comparison with the lactococcal prophages in Kelleher et al. [13] showed D6867, D7138, and D7893 to be most closely related to phage bIL309, belonging to morphotype IB; analyses showed that representative phage D6867 and bIL309 share deduced pairwise amino acid identities of ~52% for encoded Tal-RBPs and ~51% for encoded Dit proteins. Additionally, fused Tal-RBPs are found exclusively in morphotype I and V phages [13]. Based on these comparisons, we classified D6867, D7138, and D7893 as P335 morphotype IB phages [13].

**Figure 3:**
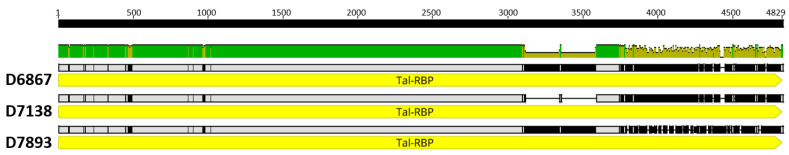
Nucleotide alignment of the genes encoding the Tal-RBPs. In the consensus identity bar (top), green indicates identity, yellow indicates polymorphism, and red indicates low identity. Yellow arrows indicate CDS. Black areas in the grey bars indicate differences among the three phages.

To identify the cause of infectivity of D7893 on the *yccB* BIMs, additional genomic analyses were performed. Comparison of D7893 against D6867 and D7138 showed a considerable level of diversity among the 3′ ends of the genes encoding the fused Tal-RBP proteins (Figure 3). The structural regions in D7893 were otherwise conserved with those of D6867 and D7138, as were the DNA packaging regions. Other notable regions where D7893 differed from D6867 and D7138 included a ~4 kb region spanning two transcriptional regulators, a host nuclease inhibitor, a DNA repair protein, an ssDNA binding protein, and two hypothetical proteins (Figure 2) and a truncated transcriptional regulator upstream of that 4 kb region.

Because Tal-RBP is involved in host recognition, attachment, and phage DNA injection [13,17,20], investigation of this region in D7893 was prioritized. The 3′ 1 kb of the gene encoding the D7893 Tal-RBP only shares ~47% pairwise nucleotide identity with those of D6867 and D7138. However, a notable difference was also identified in D7138, which harbors a 462-nucleotide in-frame deletion in a region that is conserved between D6867 and D7893 (Figure 3).

To determine if the D7893 Tal-RBP is responsible for virulence on the *yccB* BIMs, D6867 was engineered to encode the 3′ 1 kb of the gene encoding the D7893 Tal-RBP; D6867 was propagated on DGCC12699 + pG9RBP_7893_, which contains the D7893 Tal-RBP region of interest cloned on a vector with flanking regions of D6867 homology suitable for recombination. Resultant lysate was plated on a lawn of BIM2, from which plaques were observed after overnight incubation. D6867 was plated on BIM2 in parallel, which did not result in plaque formation, confirming recombination with the host prophage sequences was not responsible for infectivity. Sequencing confirmed successful integration of the D7893 Tal-RBP region of interest into D6867. A representative plaque (D6867-RBP_7893_) was propagated on BIM2 and then assayed on DGCC12699 and BIM2. Results showed the recombinant phage to infect DGCC12699 equivalently to BIM2 (Table 4). These results demonstrated that the D7893 Tal-RBP was responsible for infectivity on BIM2.

**Figure 4:**
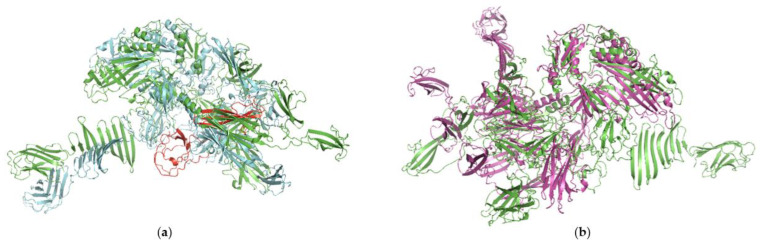
Molecular modeling and comparisons. Structural alignment of the AlphaFold predicted Tal-RBP models for: (**a**) Phages D6867 (green) and D7138 (cyan). The region representing the deletion in D7138 is shown in red on D6867; (**b**) Phages D6867 (green) and D7893 (purple).

At the deduced amino acid level, the Tal-RBP from D7893 shares ~86% and ~76% pairwise identity with that of D6867 and D7138, respectively, while the Tal-RBPs of D6867 and D7138 share ~90% pairwise identity. An HHPred protein analysis showed that the unique C-terminal end of the D7983 Tal-RBP includes the RBP domain (2FSD_A), while the Tal domain (6V8I_CE) and carbohydrate binding motifs (CBMs) (5E7T_B) were identified in the conserved regions among the three phages. Tal-RBP predicted structures were compared to evaluate potential domain level alterations that could be tied to the virulence of D7893 on *yccB* BIMs (Figure 4). Despite a TM-Score of 0.413, topological similarities were observed between D6867 and D7138 that show conservation of overall structure; the deletion in D7138 is apparently located in a relatively inaccessible region of the structure. While comparisons with D7893 reported a comparable similarity score of 0.3908, a total disruption of the overall structure was observed. In contrast to D7138, even individual domains lack significant conservation independently of orientation. Ultimately, these differences did not identify a specific region of the structure responsible for the infectivity of D7893 on the *yccB* BIMs, but that the behavior is potentially linked to a complete perturbation of the overall topology.

### 3.4. yccB–cwps Relationship

**Figure 5:**
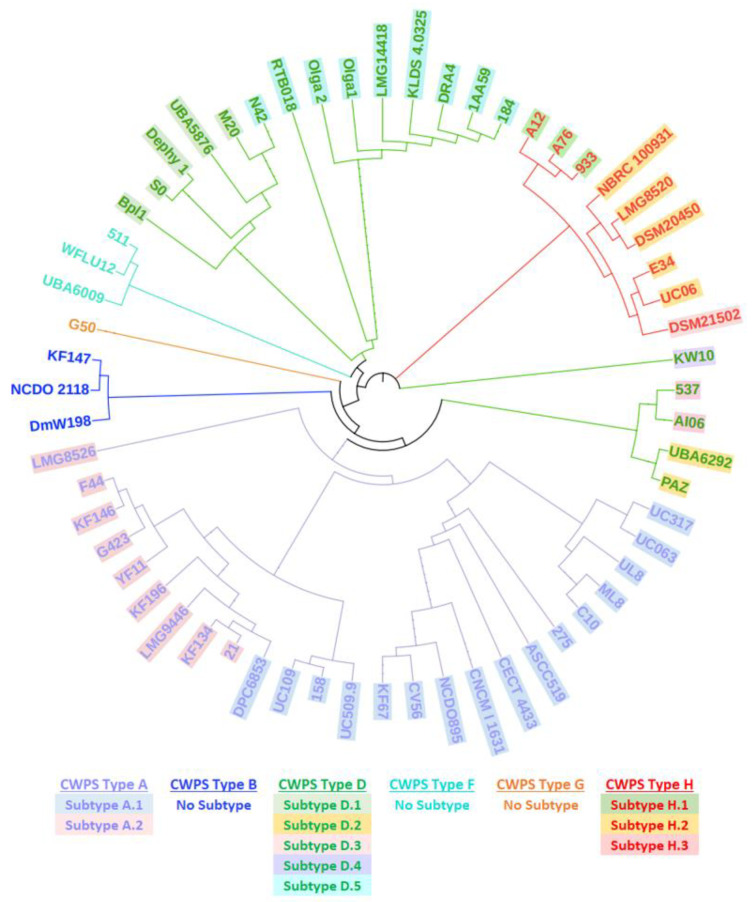
Comparison of *yccB* homologs and CWPS type. A phylogenetic tree of *yccB* homologs found in a subset of public lactococcal strains; branch color indicates CWPS type and text color/highlight indicates CWPS subtype. Like-colored strains clustering together indicate a strong relationship between *yccB* homolog and CWPS type/subtype.

To determine the distribution of *yccB* among lactococcal strains, all publicly available and internal IFF lactococcal sequences were screened for the DGCC12699 *yccB* nucleotide sequence. This analysis showed that *yccB* homologs are widely distributed among *L. lactis* and *L. cremoris*, ranging in length from 513 to 2091 bp. Interestingly, the *yccB* homologs were found in the same genetic context in all strains: immediately downstream of the *cwps* operon. Phylogenetic trees of the *yccB* homologs were created from public lactococcal strains, and the branches and text of each strain name were manually color-coded based on the *cwps* group and subgroup [2], respectively, of that strain (Figure 5 and Figure S2). Phylogenetically, a strong but not complete relationship between the *yccB* homolog and the *cwps* group/subgroup of each strain was observed. For example, CWPS type D subtypes were split between different internal branches (Figure 5). Further protein analysis on representative members of each CWPS type showed that all CWPS types, and all but one CWPS subtype, are associated with a *yccB* homolog containing a GH25 domain. Additionally, only three of the eight CWPS types are associated with a *yccB* homolog containing a YjdB domain (CWPS types A.1, A.2, D.2, D.3, and F). For reference, DGCC12699 harbors CWPS type A.1.

**Figure 6:**
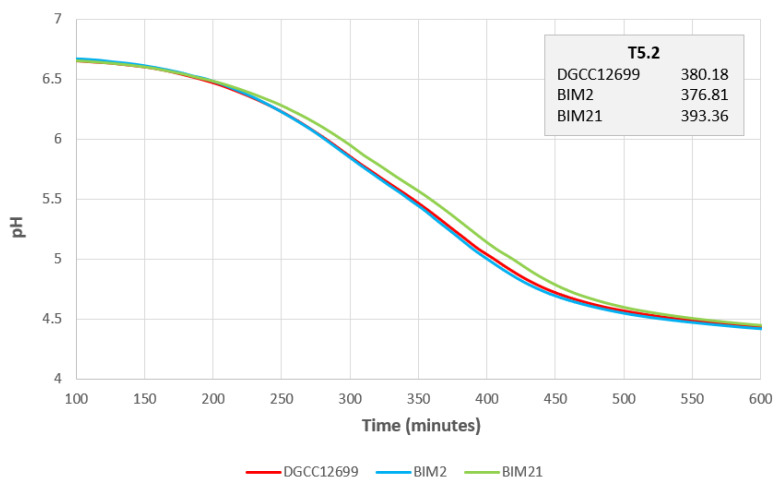
CINAC milk acidification activity test comparison of DGCC12699, BIM2, and BIM21. Each strain was run in duplicate and the averages of the data are shown on the plot. The average time for each strain to reach target pH 5.2 (T5.2) is displayed in the upper right.

Lactococcal mutants deficient in CWPS have been shown to display growth defects [2]. Therefore, milk acidification of parental strain DGCC12699, *yccb* mutant BIM2, and CWPS mutant BIM21 were compared. CINAC activity curves show BIM21 acidification properties to be notably slower than DGCC12699 and BIM2 (Figure 6). These results suggest that the *yccB* mutation may not impact CWPS production, or if it does impact CWPS, its effect does not result in growth defects under the conditions tested. This also further demonstrates that the mechanism behind the *yccB* mutation-mediated phage resistance is likely distinct from CWPS mutation-mediated resistance.

## 4. Discussion

In this study, BIMs of dairy lactococcal starter strain DGCC12699 resistant against P335-like phages D6867 and D7138 were isolated and characterized, from which two distinct phage resistance phenotypes were observed, with each correlated with a distinct mutation. BIM2, BIM8, and BIM19 were found to be mutated in *yccB,* which encodes a putative autolysin. Interestingly, mutation of *yccB* has not been previously linked to phage resistance. Complementation with the wild-type *yccB* in a representative *yccB* mutant resulted in the strain becoming fully sensitive to the P335-like phages, and subsequent curing of the cloned wild-type *yccB* restored phage resistance. Collectively, these results confirm the *yccB* mutation is responsible for phage resistance. Bioinformatic analysis showed *yccB* to harbor superfamily domains GH25 and YjdB; all three *yccB* BIMs encoded distinct mutations truncating the YjdB domain exclusively. BlastX and UniProt analyses showed GH25 to be a muramidase that hydrolyzes linkages between N-acetylmuramic acid and N-acetyl-D-glucosamine residues, aiding in cell wall biogenesis. Additional UniProt analysis showed YjdB (COG5492) to be a conserved, uncharacterized bacterial surface protein containing an Ig-like domain.

Genomic analyses of phages D6867 and D7138 showed they share a high level of nucleotide identity with each other as well as with two prophages in the DGCC12699 chromosome, indicating they may have existed in a lysogenic state before switching to a lytic life cycle or vice versa. Comparison against the P335-like prophages from Kelleher et al. [13] showed that the adhesion module in the three phages share highest amino acid identity and organizational similarity with bIL309. Based on these data, we categorized these phages as belonging to P335-like morphotype IB [13]. Phage D7893 was isolated from an industrial whey sample following commercial replacement of DGCC12699 with BIM2; analysis showed D7893 to share high nucleotide similarity with D6867 and D7138, suggesting it potentially evolved from the D6867/D7138 population in response to the *yccB* mutation. Genomic comparisons identified variation in the 3′ end of the gene encoding the Tal-RBP of D7893. Development of a recombinant phage showed that integration of this unique D7893 Tal-RBP region into the D6867 genome resulted in infectivity on BIM2. Fused Tal-RBP, exclusive to P335 morphotype I and V phages, is a fusion of two phage adhesion modules known to be responsible for host attachment and DNA injection [13,17,20]. A protein analysis performed using HHPred showed this variable region of Tal-RBP encompasses the RBP domain while the Tal and 5E7T carbohydrate binding domains remain unaffected. Phage-encoded RBP is vital in the phage infection process as it recognizes the receptor on the host cell surface [52]. Therefore, the differences identified in the RBP domains suggest the YjdB domain in YccB may affect or act as a surface receptor for D6867 and D7138. YjdB contains an Ig-like domain, and such domains are often found in cell surface proteins and have been shown to facilitate cell surface receptor functions [53]. This supports the possibility that YccB is a surface protein that functions as a phage receptor. Additionally, a review of Ig-like domains in *E. coli* revealed their presence in a diverse set of proteins, including enzymes with oxidoreductase and hydrolytic activities, ABC transporters, and sugar-binding and metal-binding proteins [54]. This diverse functionality suggests the possibility that YjdB could instead be affecting the phage receptor, for example, modifying the CWPS structure.

Interestingly, adsorption assays showed that D6867 and D7138 still bind to the *yccB* BIMs at a slightly reduced but high efficiency. It has been recently shown in lactococcal phages belonging to the *Skunavirus* genus that in addition to the RBP, CBMs contributing to phage adsorption have been found to decorate various phage structural components, including Dit, the neck passage structures, and major tail proteins [55,56]. HHPred analysis of the Dit protein, which is highly conserved among the P335 phages in this study, yielded top domain hit 5LY8_A: a tail component domain containing a CBM. Since both the 5E7T and 5LY8_A domains are conserved among D6867, D7138, and D7893, it is possible that these CBMs allow D6867/D7138 to attach to the *yccB* BIMs, however, the altered host YccB protein coupled with structural differences of the RBP region prevent complete binding of D6867/D7138, resulting in a slightly reduced adsorption efficiency and furthermore preventing phage DNA release/injection into the host cell. Contrary to autolysin LytA, which was previously shown to facilitate bacterial lysis and phage progeny release [33], our data indicate that the *yccB* mutation-mediated resistance disrupts the phage infection process earlier in the lytic cycle.

Notably, *yccB* is encoded adjacent to the *cwps* gene cluster. This gene cluster is involved in CWPS synthesis, and CWPS has been previously shown to serve as a receptor for lactococcal phages, including those belonging to the P335 group and *Teubervirus* genus [21,57]. Bioinformatic analysis of available lactococcal genome sequences revealed that *yccB* homologs can be found broadly among lactococci, and the different homologs appear to be strongly correlated with *cwps* type and subtype. This suggests a possible connection between *yccB* and CWPS; it has been previously reported that type B *cwps* transcript continues into *yccB* in *L. lactis* IL1403 [16], however, *yccB* is transcribed in the opposite direction and is separated from *tagD1* by a hairpin-loop structure. As hairpin-loop structures can function as termination sites [58], this structure likely terminates the *cwps* operon between *tagD* and *yccB*. Additionally, BIM21, which harbors a mutation in a putative glycosyltransferase within the *cwps* cluster, showed notable phenotypic differences from the *yccB* mutants, including additional resistance against D7893 and *Teubervirus* D5694, an appreciably reduced D6867 adsorption efficiency, and a slower acidification profile. Collectively, these results support that the mechanism behind the *yccB* mutation-mediated phage resistance is distinct from CWPS mutation-mediated resistance, and we hypothesize that D7893 utilizes CWPS as the primary receptor, whereas D6867/D7138 utilizes YjdB as either the primary receptor or an essential secondary receptor.

Phages are frequently problematic for lactococcal starter strains used in the dairy industry, disrupting the fermentation process and resulting in decreased product quality and economic loss. A standard approach to combat phage issues is performing natural mutagenesis on phage-sensitive starter strains to generate resistant populations [2], however, subsequent mutation(s) often disrupt genes essential for functionality in milk [3]. Thus, it is essential to continue our efforts into identifying novel phage resistance mechanisms and expanding insight into phage–host interactions. P335 species are among the most problematic in dairy environments, being remarkably diverse and difficult to address [5,6]. Due to the diversity of these phages, several distinct host-encoded genes and mechanisms have been associated with P335 resistance. This study identified a unique genetic target to address infection by some virulent P335 phages, as we have demonstrated that the wild-type putative autolysin *yccB* found in DGCC12699 is required to complete the lytic cycles of D6867 and D7138. This discovery provides insight into a novel gene involved in lactococcal phage resistance and a deeper understanding of phage–host interactions.

## 5. Patents

International Publication Number WO 2022/112284 A1 has been filed to protect the application of this study’s findings.

## Figures and Tables

**Table 2:** Primers used in this study.

Primer Name	Sequence (5′–3′)
VF-pg9	GTGGACAGAACGACACGGAT
VR-pg9	TCCTGAATCCCATTCCAGAA
CloneYccB_F	TTCTGGAATGGGATTCAGGACTACCACTATTCCCAAAGCATTTC
CloneYccB_R	ATCCGTGTCGTTCTGTCCACGCAATGGGAGATGATTGGAGAGG
RSF	TTCTGGAATGGGATTCAGGA
GC pG9vF	ATCCGTGTCGTTCTGTCCAC
D7893_TalCheck_F	CAATGCCACATTACCATATCC
pg9 + cm_R2	AACTAGTGGATCCCCCGGGCTG
internalErmR-F	GCTGAATCGAGACTTGAGTG
internalErmR-R	GTCATCTATTCAACTTATCG
yccBNested_F	GCCAACTTTGTCTTCATACTCACC
yccBNested_R	GTATGTTTTGGAAGCCCTCAGG
yccB_F1	CTCACATTATTTCACTAATACTCTTTTCTGG
yccB _F2	CATGGATTGGAACAGATACAGCAACTTG
yccB _F3	CAATACGATCTATACTACCGAGTTCAAGC
D6867tal_StartF	GGAAGCTGGTGGTCAGATTTG
D6867tal_F1	CCAATACTGAGCCGACCACTC
D6867tal_EndR	GACCCATTCGTGAAGTCATTG

**Table 3:** Hi-Fi assembly constructs.

		pG9yccB	pG9RBP_7893_
Vector fragment	Forward primer	VF-pg9	VF-pg9
	Reverse primer	VR-pg9	VR-pg9
	Template	pG9	pG9
Insert fragment	Forward primer	CloneYccB_F	D7893_RBP-Rec (gBlock)
	Reverse primer	CloneYccB_R	
	Template	DGCC12699	
Detection of TG1 RepA+ transformants	Forward primer	CloneYccB_F	D7893_TalCheck_F
	Reverse primer	CloneYccB_R	pg9 + cmR_2
Detection of L. lactis transformants	Forward primer	RSF	D7893_TalCheck_F
	Reverse primer	GC pG9vF	pg9 + cm_R2

**Table 4:** Phage assays. EOP is the average of three independent replicates ± sample standard deviation. When no plaques/phage inhibition were visible, EOP is < the highest value of three independent replicates. Hyphens indicate the combination was not tested.

	EOPs					D6867 Adsorption (%)
	D6867	D7138	D7893	D5694	D6867-RBP_7893_	
DGCC12699	1	1	1	1	1.03 ± 0.08	97.4 ± 1.3
BIM2	<9.09 × 10^−8^	<2.00 × 10^−7^	0.75 ± 0.15	1.31 ± 0.20	1	82.3 ± 2.9
BIM8	<9.09 × 10^−8^	<2.00 × 10^−7^	0.82 ± 0.19	1.16 ± 0.41	--	87.8 ± 3.2
BIM19	<9.09 × 10^−8^	<2.00 × 10^−7^	0.78 ± 0.18	0.91 ± 0.22	--	87.5 ± 3.4
BIM21	<9.09 × 10^−8^	<2.00 × 10^−7^	<3.33 × 10^−6^	1.02 × 10^−3^ ± 7.76 × 10^−4^	--	41.1 ± 4.4
BIM2 + pG9yccB	1.20 ± 0.20	0.98 ± 0.08	--	--	--	--
BIM2 + pG9	<9.09 × 10^−8^	<2.00 × 10^−7^	--	--	--	--
BIM2ΔpG9yccB	<9.09 × 10^−8^	<2.00 × 10^−7^	--	--	--	--

## Data Availability

The DGCC12699 *yccB* gene sequence and complete genome sequences of phages D6867, D7138, and D7893 are available at the NIH GenBank database (www.ncbi.nlm.nih.gov/genbank, accessed on 12 September 2023). Accession numbers are as follows: DGCC12699 *yccB*: OR544030, D6867: OR480986, D7138: OR480987, D7893: OR480988.

## Supplementary Materials

The following supporting information can be downloaded at: https://www.mdpi.com/article/10.3390/v15112193/s1, Figure S1: Tal-RBP exchange by homologous recombination; Figure S2: Additional comparison of autolysin homologs and CWPS type.

## Appendix A

**Table A1:** gBlock gene fragment sequence.

gBlock Name	Sequence (5′–3′)
D7893_RBP-Rec	TTCTGGAATGGGATTCAGGAACAACGGATAATCCACCTAAGGTTGGAGATACCATTATTGAT TTGGTAGGTAAGATTTATCAGATTACCAATGTAGTAGTTGATAGTACAATAGGTGGCGGCGG TCTATTCGACTATGGCCCAATGCTTACAAGTATCAAAGGCGCAGATGGTTCGCCGGGTAAG GACGGTATCGCTGGAAAAGATGGTGTTGGATTAAAAAGCACTATTATTACTTATGGGATTTC AGCATCAGATAGTACCATGCCGGGGACGTGGTCTCAAAATACACCAGCATTAGTTAAGGGA CAATGGTTCTGGACTAAAACACAGTGGACCTATACAGATAACAGCACTGAAACTGGTTATC AAAAAACATATATCTCTAAAGACGGAAATAACGGTCATGACGGAATTGCTGGTAAAGATG GTACTGGAATCAAAACTACGACCATTACATACGCAGGTTCAACAAGCGGAACGACAGCAC CAAATACTGGTTGGACTACCACAGTTCCGACAGTTGCAGCAGGTAATTACCTGTGGACTAA GACTGTTTGGGATTATACGGATAAGACCAGCGAGACAGGTTATTCAGTAGCTAAAATGGGA AATAATGGAACACCTGGTCCGCAAGGTCCTCCTGGAAGTAATGGTGATCCTGGTAAAACTG TTTCCAATACTGAGCCGACCACTCGATTTAAAGGCTTGACTTGGAAATACTCAGGTACGACT GACCTTACAGCGAGTGATAGAACAGTGATTAAGCCAAATACAGAGTATTACTATAATGGCA CTCATTGGGTGATTAACTATTTTAGCGTCAATAACTTTGCGGCTGAATCGATAACATCAGAT AAAATTGATGGTAAAAATTTAACAATTACTGATGGTGAGTTCATAAGCAAAACATCTAATG GCCCAGTTACAACCTCTACAGAAATCAAAGATAATCATATTGCAATTTCAAAGAAAGATGG AACTGTTAATACTAGAAATGATATAGCGCTTGATTCTGAACAAGGACTAGCTCAGAAATTTA CGAACATTAATACAGGATTCTACAGAACAGCTGGGATTAATTATCAAGGTCCATTCACAAG TGACTCAGATGGAAACTATGCTCAACTTACACCTCAAGGCACGAAGTTATCTACCGATGTTC CTTGGACCAAGCTTAGTTTGATGAATAATTTTAATGGAAATATTGAGTATGCAATTATCAAT GGGACTGTCTATATATCAGCGTCAGGAGTTGGTGTACCAGCAATGACTGCTGGTCAATGGA AGCAAGCGGCTCAATTGCCAACAGGAAGTTCAGCAATTCCAATTAGAGCAAATCGAATTGC AGCAGGAGATAGTGGAGATGGTCTAAGTTGGGCATTACTTTCTAATCAGGCTGGAGGAATA TTCATTCGATGCAGTGCTAATAAAGCACCAACACCTAACTTATTCAATGCCACATTACCATA TCCAATAGGATAAAAGGAGGAAAAATGGAAAAAGTCAATACAACGAATACAACAACTGAT ATCTTTGTCGGTGATAAGAATGTGGGTAATTTCACCCTTACAACGTTCAATAACGGAACAAT GAATGCAAATTTCATGATTAATGACCCTACAGCATTTCATGGCACGCCAGAAGCAGCTCAA GACCTAGCTAATTTGGTTAGCTCGGCAGTTAATCAGTCTAAAGCTTTGTTGGCTGATTTTGAA GCTAGTAAAGAATAGAAAGCAGGGGTTATGGAATTAGAACAACTTGTGGAACAGCATGAG GACAAACTCAAGCGGCACGATAAAGAATTATCTCGACTTAATGATATGTCAGTCGAAATAC AAAAGCAAATGAATGACGGATTGACTCGTGTGGATGAATCCAATCGCTTTTTAAGAGAACA GAATACTCGACAATCTGAACAGAATGCTCAAATACTACAAGCAGTTATCAAAGGTAATGAA AGCTCAGACGAAGTGGACAGAACGACACGGAT
