# Supplementary material for: Novel P335-like Phage Resistance Arises from Deletion within Putative Autolysin yccB in Lactococcus lactis
Source: Viruses. 2023 Oct 31;15(11):2193. doi: 10.3390/v15112193 (PMC10675428; doi:10.3390/v15112193)
Supplement: Supplementary file 1 [file viruses-15-02193-s001.zip › viruses-2663649-supplementary.pdf]

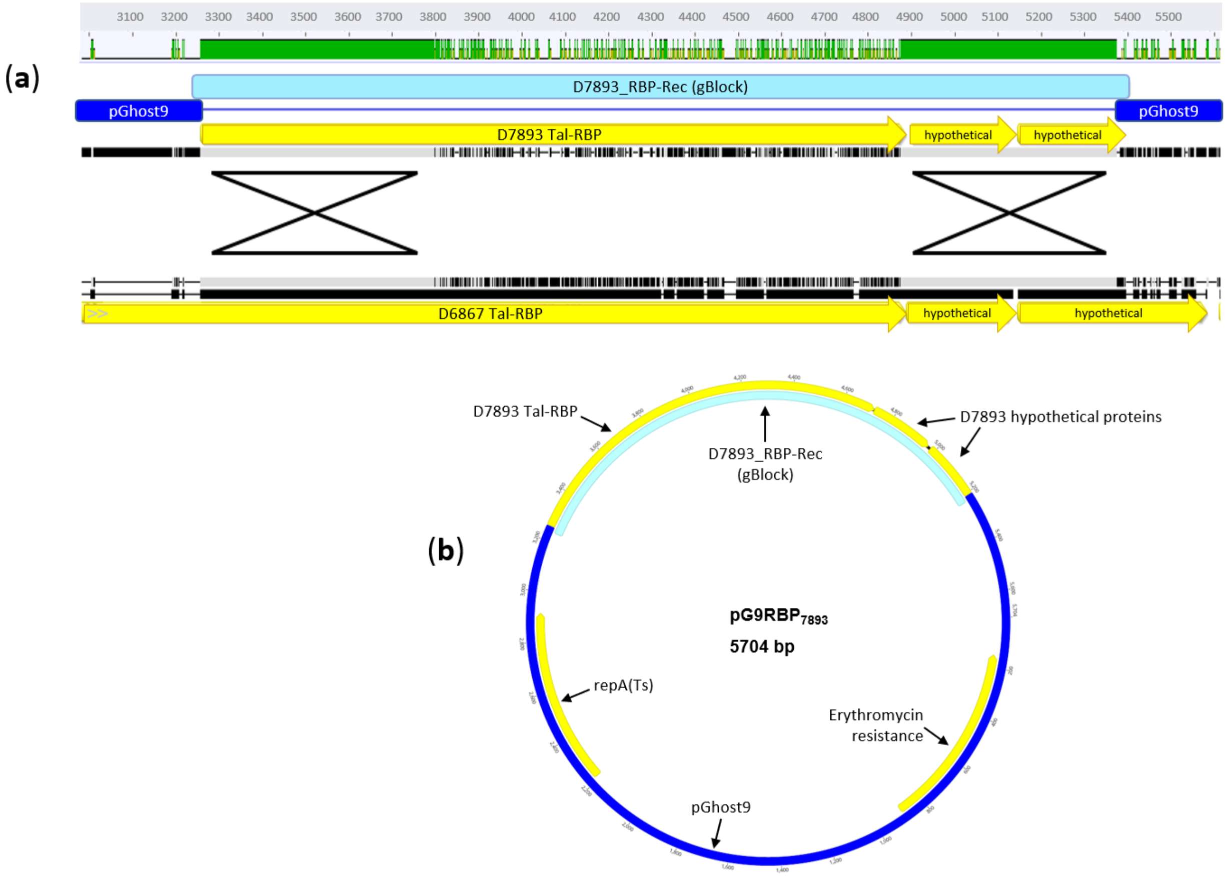

Figure S1: Tal-RBP exchange by homologous recombination. (**a**) DNA sequence alignment of the D7893 *tal-rbp* cloned onto pG9 and *tal-rbp* encoded by phage D6867, including flanking regions. (**b**) pG9RBP$_{7893}$ vector map. Dark blue bars indicate pGhost9 sequence. Yellow arrows indicate CDS. Light blue bars indicate gBlock DNA fragment. In the consensus identity bar, green indicates identity, yellow indicates polymorphism, and red indicates low identity. The sequence homology found at the 5′ and 3′ ends of the alignments served as regions for homologous recombination.

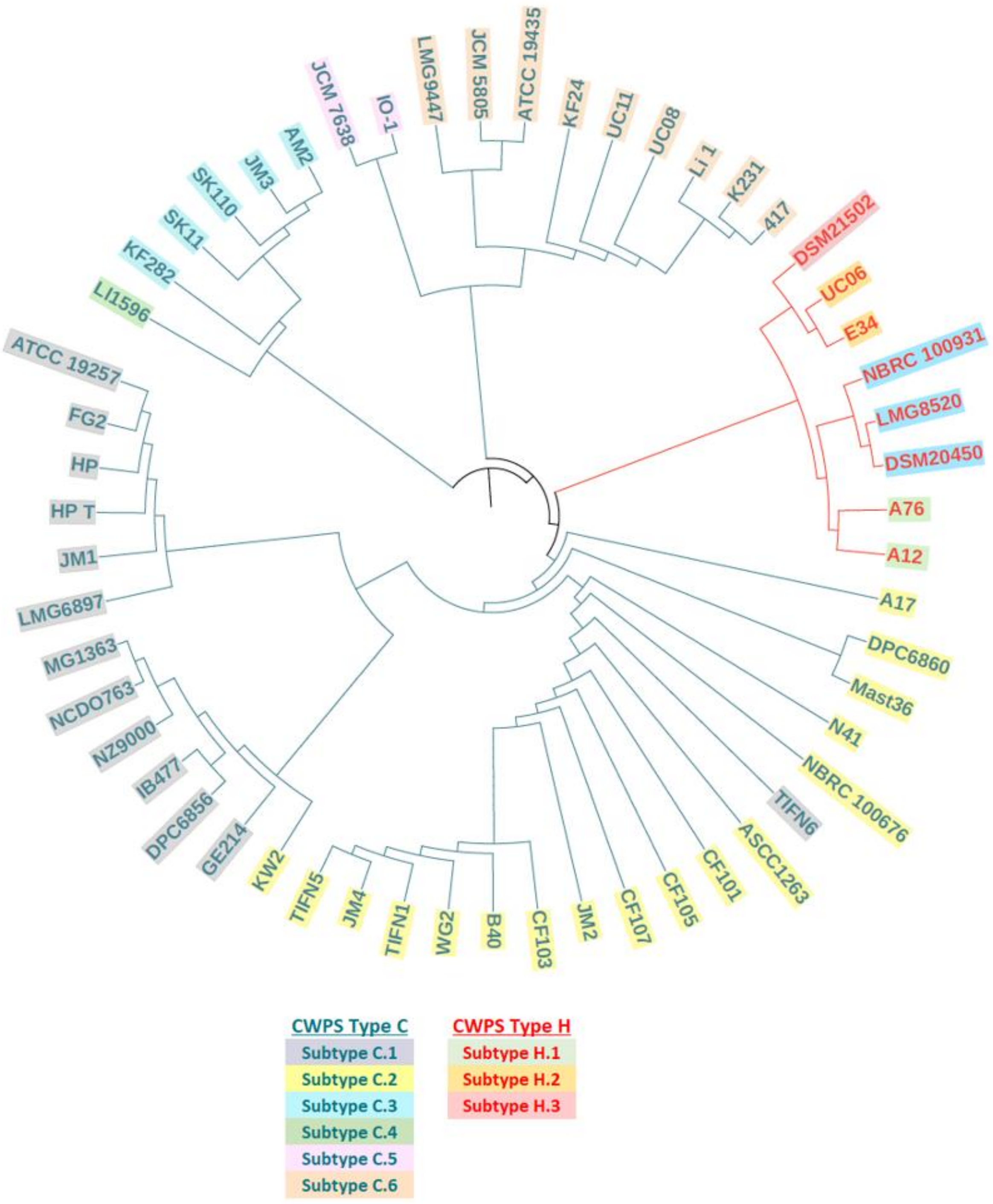

Figure S2: Additional comparison of *yccB* homologs and CWPS type. A phylogenetic tree of *yccB* homologs found in a subset of public lactococcal strains; branch color indicates CWPS type and text color/highlight indicate CWPS subtype. Like-colored strains clustering together indicate a strong relationship between *yccB* homolog and CWPS type/subtype.
